# The Power of the Claw

**DOI:** 10.1371/journal.pone.0073811

**Published:** 2013-09-04

**Authors:** Bruce M. Rothschild, Bill Bryant, Christopher Hubbard, Kent Tuxhorn, Ginny Penn Kilgore, Larry Martin, Virginia Naples

**Affiliations:** 1 Biodiversity Institute, University of Kansas, Lawrence, Kansas, United States of America; 2 Northeast Ohio Medical University, Rootstown, Ohio, United States of America; 3 Sedgwick County Zoo, Wichita, Kansas, United States of America; 4 Department of Biological Sciences, Northern Illinois University, DeKalb, Illinois, United States of America; University of Utah, United States of America

## Abstract

Scratches on bones have routinely been attributed to tooth marks (a predominantly untested speculation), ignoring the effects of claws, perhaps because of the general assumption that claws are too soft to damage bone. However, some pathologies appears to be more compatible with claw rather than tooth impacts. Therefore, it is critical to determine if the claws of any animal are capable of scratching into the surface of any bone – a test and proof of concept. A tiger enrichment program was used to document actual bone damage unequivocally caused by claws, by assuring that the tiger had access to bones only by using its paws (claws). The spectrum of mechanisms causing bone damage was expanded by evidentiary analysis of claw-induced pathology. While static studies suggested that nails/claws could not disrupt bone, specific tiger enrichment activities documented that bones were susceptible to damage from the kinetic energy effect of the striking claw. This documents an expanded differential consideration for scratch marks on bone and evidences the power of the claw.

## Introduction

Linear defects on bones have traditionally been attributed to the action of predator/conspecific or even herbivore teeth [1]–[19]. Spacing and character of scratches have been so attributed by Sutcliffe [1], Haynes [3], Eickhoff and [6], Haynes [14], Faith and Behrensmeyer [15], Montalvo, et al. [16], Muñoz et al. [17] and Dominato et al. [19] and the damage experimentally documented by Eickhoff and Herrmann [6], Haynes [5] [14], Haglund and Sorg [9] and Muñoz et al. [17]. Rothschild [20] and Sharpe [21] hypothesized that at least some of the marks on bones instead were caused by claws, but the question remains: A claw can cause a linear defect in soft tissues, but can it actually cause damage to a bone? After all, claws are not as hard as bone. The prevailing concept of hardness in geology defines it as a determinate of the ability of one structure to scratch another according to the Mohs hardness scale [22]. In this graded series, where 1 = softest (talc) and 10 = hardest (diamond), the keratin that composes claws is at 2.5 while bone is rated at 5. Therefore, bone is about twice as hard as the keratin that makes up claws. Nevertheless, small V-shaped punctures in bone have been recognized that are the result of bird talons [23]. There have also been and fictional accounts where scratches from claws were mentioned [24].

Perhaps the effect of kinetic energy at the impact site and the properties of the impacting structure ought to be examined. Ojeda and colleagues [25] provided an example of a relevant phenomenon. Remains of a 30–40 year old woman (apparently a shaman or healer) from the Central California CCo-295 site, dated at 2500–3500 years before present, showed multiple fractures from a crushing injury. A phalanx from the *Ursus* (bear) paw elements, that she was wearing at the time, had been driven into the supra-articular region of her humerus. These examples suggest that both bird and mammal claws can produce puncture wounds, but we must also demonstrate that they also can cause scratches.

While theoretical approaches are important, medical approaches to disease and trauma are often empirical – i.e., in vivo veritas. Therefore, it is reasonable to pursue an experimental assessment of the ability of claws to affect bone. Rather than use a mechanical model, we elected to use an ecological assessment. The effect of carnivore claws on herbivore bones seems generalizable, as the surface response of bone to injury has been documented as independent of phylogeny, at least between reptiles and mammals, including humans [26]. Both have circumferential laminae parallel to the periosteal surface [27]–[29]. The current analysis addresses a previously unrecognized source of bone scratches, affording a new window to attacking, killing and feeding behaviors.

Two components are required if we are to assess successfully the ability of claws to cause damage to bones. The first requirement is to assure that the target bone is accessible only to the claws of the predator and that it cannot be reached by the teeth of the foraging animal. The second aspect is verification that the animal actually attacked the bone. Making this assessment was feasible by studying a tiger (*Panthera tigris*) at the Sedgwick County Zoo (Wichita, Kansas). Our experiment was incorporated into the zoo's tiger enrichment program, wherein animal behavior was monitored. The goal was to determine whether active use of claws would result in recognizable bone damage. That goal was achieved.

## Materials and Methods

All animal work was conducted according to relevant national and international guidelines, as part of an animal enrichment program, as was approved by the Animal Care Committee of the Sedgwick County Zoo, Wichita, KS.

**Figure 1:**
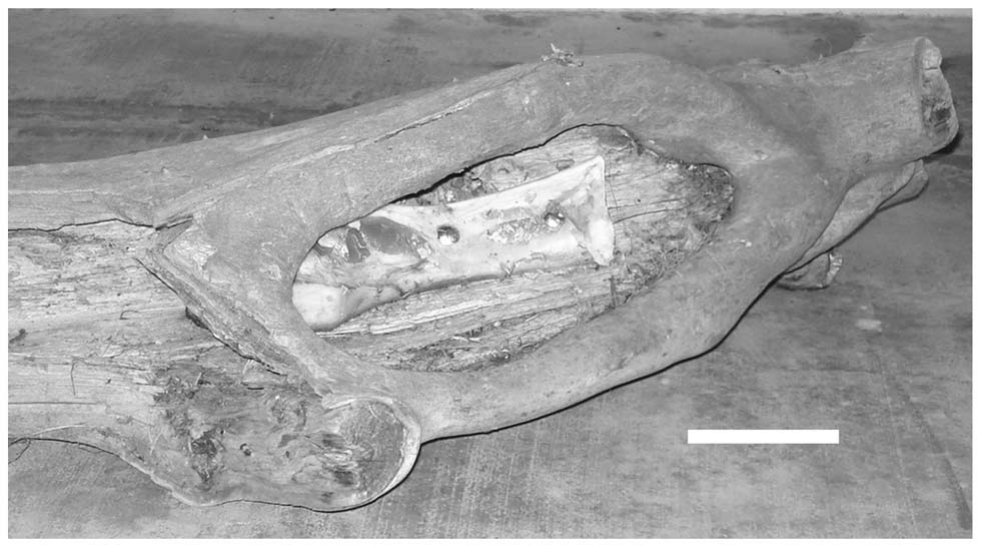
Bovid femur bolted in log, accessible to paws, but not jaws. Bar equals 10 cm.

Soft tissues were carefully removed from cow femora (curated in the skeletal collection of the Sedgwick County Zoo), taking care not to contact the periosteal surface. The bones were examined carefully to assure that none had been altered prior to their use in the experiment. This was done to assure that damage noted subsequent to the tiger interaction was not from the process of defleshing the bones prior to damage assessment. Two bones were positioned such that they could not be mouthed, but only accessed by the claws of the tiger. A small opening was made in a hollowed-out log, and the cow femora bolted inside (Fig. 1). The hole (both before and after enrichment activities) was verified as being too small to admit entry of the animal's snout. The tiger expressed major interest in the object, pawing at the bone in the log, but was unable remove it from its bolted location. After the enrichment activity was completed, the log was removed from the tiger habitat. At that time, the bone was unbolted from its position and examined macroscopically, including rotating the bone to changing the incident light angle [30]. Sites showing claw induced alterations were subsequently examined and photographed using a dissecting microscope (Wild M3C dissecting photomicroscope). Any surface alterations were subsequently sectioned by band saw. Two samples taken from the damaged area were demineralized and sectioned for analysis using a light microscope. Three other specimens were processed for scanning electron microscopy (JEOL 56101v Environmental SEM).

## Results

**Figure 2:**
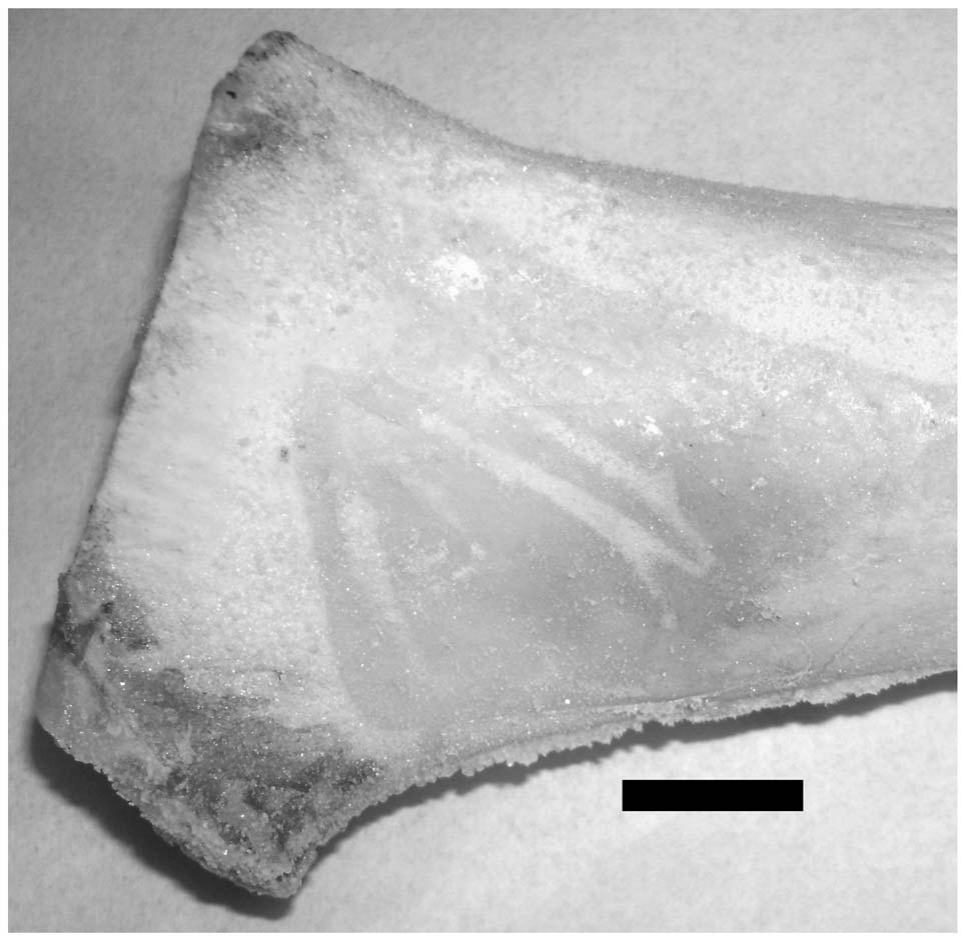
Macroscopic view of scratches. Documentation of claw-produced scratches. Bar equals 5 cm.

**Figure 3:**
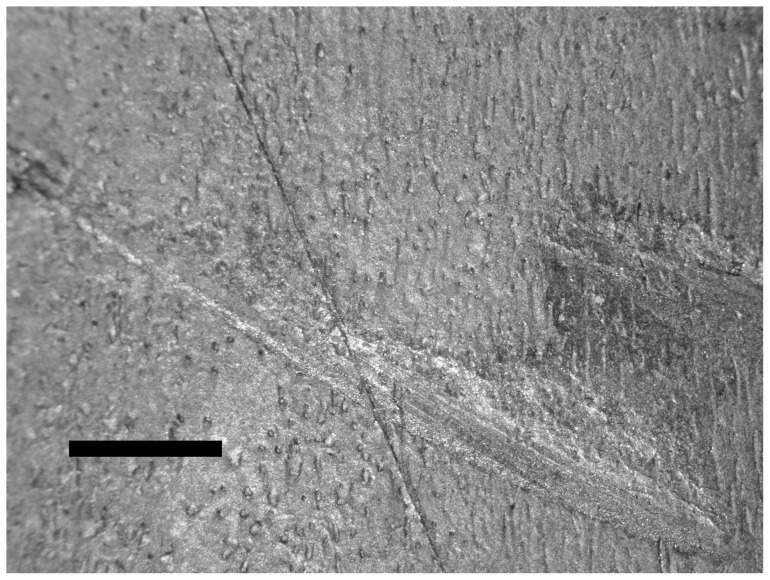
Low power microscopic view of several scratches on the bone surface. Bar equals 1 cm.

**Figure 4:**
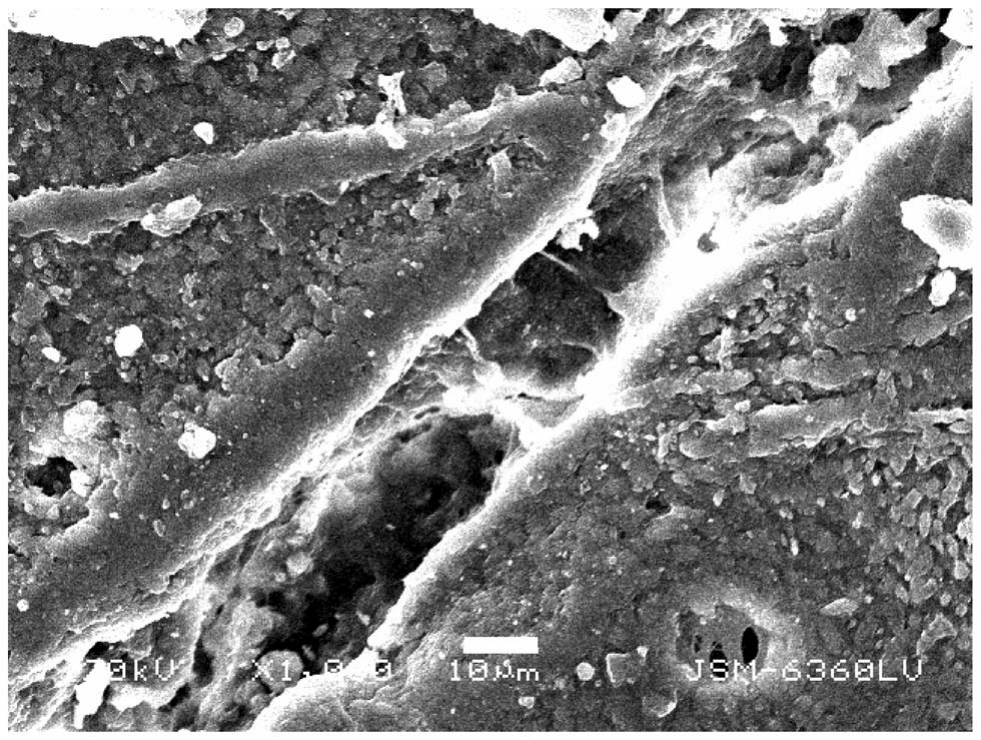
Scanning electron microscopic image of shallow gouge produced by tiger claw. Bar equals 10 microns.

Gross macroscopic examination of the bone surface revealed four scratches (Fig. 2). Stereo-microscopic examination revealed a diagonal cut had been produced and that it had penetrated the periosteum and subjacent bone (Fig. 3). Scanning electron microscopic examination of the thin scratch in the region of the shallow gouge documented that the scratch penetrated the bony matrix (Fig. 4). The bony surface surrounding the scratch appeared marred and lacked periosteal covering when compared to unscratched (control) regions that retained an intact periosteal covering.

## Discussion

Haglund [31] on page 411 reported that claw-induced, linear, “scratch-type abrasions” could be produced in soft tissues by carnivores. This study documents that their effect is not limited to soft tissues. Claws have their origin in elongation of terminal scales [32]–[35]. Their capabilities are now documented as not limited to producing puncture damage to bone [20]. Clearly, claws can produce bone damage, documenting that structural hardness is not the only determinant of whether contact between two structures causes damage to the one perceived as being of greater hardness. Kinetic energy of interaction is an important consideration, as has even been documented for teeth. Meers [33] on page 2 noted that penetration of teeth is “resisted by the properties of the flesh (fiber density and strength of dermal armor, if present)” and by the “friction between the surface areas of the teeth and flesh.” Damage by claws on bone appears to represent an analogous phenomenon. Sufficient kinetic energy of the claw strike can penetrate soft tissue with enough energy for the claw to penetrate bone, as has been documented for velocity-driven softer objects penetrating hard structures [36]–[40]. Scratches can also disrupt periosteum at a young age, allowing the resultant organism/bone growth to produce increased separation of the edges. The defect could then become even more prominent as the individual ages.

Haglund and Sorg [9] an page 14 write that “perspective shifts are common sequelae when one field of study is applied in a different disciplinary context.” Such is the case with this analysis. It utilized an enrichment program for a zoo animal to extend the range of attributions regarding their origins when scratches on bone are detected. Assessment of bone alterations should consider the impact of claws and nails, as well as bites.

## References

[1] Sutcliffe A (1971) Similarity of bones and antlers gnawed by deer to human artifacts. Nature 246: 428–430. 10.1038/246428a0 4587155

[2] Bonnichsen R (1973) Some operational aspects of human and animal bone alteration. In: Gilbert M, editor. Mammalian Osteo-Archaeology: North America. Columbia, Missouri: Missouri Archeol Soc. 9–24.

[3] Haynes G (1980) Prey bones and predators: Potential ecologic information from analysis of bone sites. Ossa (1980): 775–797.

[4] Haynes G (1982) Utilization and skeletal disturbances of North American Prey carcasses. Arctic 35: 226–281.

[5] Haynes G (1983) A guide for differentiating mammalian carnivore taxa responsible for gnaw damage to herbivore limb bones. Paleobiol 9: 164–172.

[6] Eickhoff S, Herrmann B (1985) Surface marks on bones from Neolithic Collective Grave (Odagsen, Saxony): A study on differential diagnostics. J Hum Evol 14: 263–274.

[7] Tanke DH, Currie PJ (1995) Intraspecific fighting behavior inferred from toothmark trauma on skulls and teeth of large carnosaurs (dinosaurs). J Vert Paleontol 15(3, Suppl): 55A.

[8] Haglund WD (1996) Dogs and coyotes: Postmortem involvement with human remains. In: Haglund WD, Sorg MH, editors. Forensic Taphonomy: The Postmortem Fate of Human Remains: Danvers, Massachusetts: CRC Press. 367–382.

[9] Haglund WD, Sorg MH (1996) Method and theory of forensic taphonomic research. In: Haglund WD, Sorg MH, editors. Forensic Taphonomy: The Postmortem Fate of Human Remains: Danvers, Massachusetts: CRC Press. 313–326.

[10] Tanke DH, Currie PJ (1998) Head-biting behavior in theropod dinosaurs: Paleopathological evidence. Gaia 15: 167–184.

[11] Keiran M (1999) Discoveries in palaeontology – Albertosaurus – Death of a predator: Vancouver, British Columbia: Raincoast Books. 56 p.

[12] Molnar RE (2001) Theropod paleopathology: A literature survey. In: Tanke DH, Carpenter KE, editors. Mesozoic Vertebrate Life. Bloomington, Indiana: Indiana University Press. 337–363.

[13] Haglund WD, Sorg MH (2002) Advances in Forensic Taphonomy: Method, Theory, and Archaeological Perspectives. DanversMassachusetts: CRC Press. 544 p.

[14] Haynes G (2005) Las acumulaciónes modernas de huesos de elefante como modelo para interpreter Ambrona y otras areas con fauna fósil a orillas del agua. In: Santonja M, Pérez Gonzalez A, editors. Los Yacimientos Paleolîticos de Ambrona y Torralba (Soria). Alcalá de Henares, Spain: Museo Arqueológicas. 154–174.

[15] Faith JT, Behrensmeyer AK (2006) Changing patterns of carnivore modification in a landscape bone assemblage, Amboseli Park, Kenya. J Archeol Sci 33: 1718–1733.

[16] Montalvo CI, Pessio ME, González VH (2007) Taphonomic analysis of remains of mammals eaten by pumas (Puma concolor, Carnivora, Felidae) in central Argentina. J Archeol Sciv. 34: 2151–2160.

[17] Muñoz AS, Mondini M, Duran V, Gasco A (2008) Los pumas (Puma concolor) como agentes tafonómicos. Análisis actualístico de un sitio de matanza en los Andes de Mendoze, Argentina. G v os 41: 123–131.

[18] Gignac PM, Makovicky PJ, Erickson GM, Walsh RP (2010) A description of Deinonychus antirrhopus bite marks and estimates of bit force using tooth indention simulations. J Vert Paleontol 30: 1169–1177.

[19] Dominato VH, Mothé D, Costa Da Silva R, Dos Santos Avilla L (2011) Evidence of scavenging on remains of the gomphothere Haplomastodon waringi (Proboscidea: Mammalia) from the Pleistocene of Brazil: Taphonomic and paleoecological remarks. J South Amer Earth Sci 31: 171–177.

[20] Rothschild BM (2013) Clawing their way to the top: Tyrannosaurid pathology and lifestyle. In: Parish M, Molenar RE, Currie PJ, Koppelhus EB, editors. Rockford Museum Symposium on Tyrannosaurs. Indiana University Press, Bloomington, Indiana. 210–221.

[21] Sharpe K (2005) Line markings: Human or animal origin? Rock Art Res 21: 57–84.

[22] Marshak S (2004) essentials of Geology. New York and London, W. W. Norton Press. P. 89.

[23] Sanders WJ, Trapani J, Mitani JC (2003) Taphonomic aspects of crowned hawk-eagle predation on monkeys. J Hum Evol 44: 87–105. 12604306 10.1016/s0047-2484(02)00196-3

[24] Simmons D (2007) The terror. New York: Back Bay Books, Little, Brown & Co. 784 p.

[25] Ojeda HM, Richards GD, Ibarra CL, Horton CF (2001) Bear phalanx traumatically introduced into a living human: Prehistoric evidence. Amer J Phys Anthropol 2001 (Suppl): 228. 10.1016/j.ijpp.2013.01.001 29539359

[26] Rothschild BM, Martin LD (2006) Skeletal Impact of Disease. AlbuquerqueNew Mexico: New Mexico Museum of Natural History Press. 226 p.

[27] Carter DR, Hayes WC, Schurman DJ (1976,) Fatigue life of compact bone. II. Effects of microstructure and density. J Biomech 9: 211–218. 1262355 10.1016/0021-9290(76)90006-3

[28] Saha S, Hayes WC (1977) Relations between tensile impact properties and microstructure of compact bone. Calc Tissue Res 24: 65–72. 10.1007/BF02223298 597746

[29] De Ricqles AJ (1980)) Tissue structures of dinosaur bone. In: Thomas RD, Olson EC, editors. A Cold Look at Warm-Blooded Dinosaurs. Boulder, Colorado: Westview Press. 103–139.

[30] D'amore DC, Blumenschine RJ (2009) Komodo monitor (Varanus komodoensis) feeding behavior and dental function reflected through tooth marks on bone surfaces, and the application to ziphodont paleobiology. Paleobiol 35: 525–552.

[31] Haglund WD (1996) Rodents and human remains. In: Haglund WD, Sorg MH, editors. Forensic Taphonomy: The Postmortem Fate of Human Remains. Danvers, Massachusetts: CRC Press. 405–414.

[32] Hamrick MW (2001) Development and evolution of the mammalian limb: Adaptive diversification of nails, hooves and claws. Evol Develop 3: 355–363. 10.1046/j.1525-142x.2001.01032.x 11710767

[33] Meers MB (2002) Maximum bite force and prey size of Tyrannosaurus rex and their relationships to the inference of feeding behavior. Hist Biol 16: 1–12.

[34] Alibardi L (2004) Dermo-epidermal interactions in reptilian scales: Speculations on the evolution of scales, feathers and hairs. J Exp Zool 302B: 365–383. 10.1002/jez.b.20028 15287101

[35] Alibardi L (2010) Autoradiographic observations on developing and growing claws of reptiles. Acta Zool (Stockholm) 91: 233–241.

[36] Ghadiri M, Zhang Z (2002) Impact attrition of particular solids. Part 1: A theoretical model of chipping. Chemical Engineering Science 57: 3659–3669.

[37] Wang YQ, Huang LP, Liu WL, Li J (1998) The blast erosion behavior of ultrahigh molecular weight polyethylene. Wear 218: 128–133.

[38] Eivakar M, Agarwal VK, Singh SN (2005) Effect of the material surface hardness on the erosion of AISI316. Wear 259: 110–117.

[39] Melaragno M (1996) Severe Storm Engineering for Structural Design. Amsterdam: Gordon and Breach Publishers SA, p. 133.

[40] Keller D, Vonnegut B (1976) Wind speeds required to drive straws and splinters into wood. J Appl Meteorology 15: 899–901.

